# Sterilization Procedures for Titanium Alloy Surfaces Leads to Higher Expression of Biofilm-Related *Staphylococcus aureus* Genes

**DOI:** 10.3390/antibiotics11111647

**Published:** 2022-11-17

**Authors:** Christopher Spiegel, Michael Nogler, Débora C. Coraça-Huber

**Affiliations:** Research Laboratory for Biofilms and Implant Associated Infections (BIOFILM LAB), University Hospital for Orthopaedics and Traumatology, Medical University of Innsbruck, Müllerstrasse 44, Room 1.30a, 6020 Innsbruck, Austria

**Keywords:** biofilm, implant-related infections, sterilization methods, pressurized saturated steaming, UV light sterilization, *Staphylococcus aureus*

## Abstract

**Background**: Around 1–2% of all implantation surgeries lead to implant-related infections, incurring costs of $40,000–$160,000 per total hip PJI. The 5-year mortality rate of prosthetic joint infections is up to 21%. To prevent infections during surgery, sterile surgery rooms and procedures have been developed and certified standards have been established. To guarantee the sterility, implants can be acquired already sterile from manufacturers. Some titanium implants can be delivered unsterilized with a manual for sterilization procedure in compliance with ISO 17664. The aim of this study is to evaluate if the most used sterilization methods (steam sterilization in an autoclave and UV light sterilization) of titanium alloys, can influence the biofilm forming capacity of *Staphylococcus aureus*. In this study, we examined the influence of sterilization methods on the gene expression of biofilm-associated genes and regulators. **Methods**: We compared gene expression of *icaADBC*, *SarA*, *SigB*, and *SodA* on titanium CP4 and Ti6Al4V alloys sterilized by UV-light and pressurized saturated steam sterilization. We performed RT-qPCR after RNA extraction of *Staphylococcus aureus* ATCC 29213. In addition, bacterial cell growth on the sterilized titanium surfaces was examined by colony forming unit counting on agar plates after 24 h of incubation. **Results**: Colony forming units of *S. aureus* on titanium CP4 samples showed a higher tendency in colony counts when sterilized with UV light than with pressurized saturated steam (autoclaved). Similarly, colony forming unit counts on Ti6Al4V samples showed tendencies of higher numbers on UV light sterilized samples than on autoclaved samples. Gene expression of *icaADBC*, *SarA* and *SodA* between steamed samples and UV light sterilized samples showed no difference on titanium CP4 samples, whereas *SigB* showed higher gene expression on titanium CP4 samples when sterilized with UV light than in an autoclave. On autoclaved Ti6Al4V samples, all examined genes showed 4 to 9 times higher fold changes in gene expression than on UV light sterilized samples. **Conclusions:** This study indicates that steam sterilization of Ti6Al4V can increase biofilm formation of *S. aureus* on its surface. The significantly increased gene expression of biofilm responsible genes may indicate a modification of titanium surfaces on alloy components. This may promote biofilm formation that can lead to implant-infections in vivo.

## 1. Introduction

Titanium grade 4 and titanium grade 5 are widely used as implant materials in orthodontics and orthopedics. Both materials are used due to their high strength, biocompatibility, and corrosion resistance. The corrosion resistance is provided by a natural thin layer of TiO_2_ usually incorporated on the surface of implant materials [1]. Around 1–2% of all implantation surgeries experience implant-related infections, incurring costs of $40,000–$160,000 per total hip PJI [2,3]. The 5-year mortality rate is up to 21% within PJI infections [4]. Therefore, biofilm associated prosthetic joint infections (PJI) are a burden for patients and medical health systems. Many approaches to modify implant surfaces to enhance osseointegration and reduce risks for implant infection have already been investigated [1,5,6]. When used as indwelling medical devices, all biomaterials must be priorly sterilized [7]. Usually, implants are delivered to the surgeons sterilized from the manufacturer, or unsterilized with a manual for the sterilization procedure, as per ISO 17664 [8]. These sterilization procedures differ from the manufacturer’s sterilization protocols, as manufacturers mainly use gamma rays or ethylene oxide to sterilize their products. The ISO 17664 procedures include several washing steps followed by the sterilization in an autoclave at 135 °C for 15 min. Vezeau et al. compared the fibroblast adhesion on titanium surfaces sterilized by different methods. This study showed that sterilization by pressurized saturated steam and with ethylene oxide contaminated sample surfaces and led to an alteration in fibroblast adhesion [9]. Similar studies on the effect of sterilization methods on titanium surfaces and biofilm formation have not yet been performed.

*Staphylococcus epidermidis* and *Staphylococcus aureus* are the most common infecting agents causing implant-related infections due to their vast repertoire of pathogenic mechanisms. One of these mechanisms is the ability to form a biofilm. Biofilm is a defensive mechanism where bacterial cells use quorum sensing to form cell aggregations that can withstand environmental stress, lack of nutrients, or a rise in antibacterial substance concentrations. Additionally, bacterial cells increase horizontal gene transfer during biofilm formation [10]. Biofilm formation is one reason why the use of therapeutics against PJI is limited, and why a 2-stage revision is urgently needed to reduce patient mortality risk [11]. 

Several genes are responsible for biofilm formation. Bacterial cell stress activates the gene regulator *sigma B*. *Sigma B* regulates the transcriptional regulating gene *sarA* which is also regulating the formation of polysaccharide intercellular adhesin (PIA) [12]. PIA are responsible for bacterial cell linkage in between the biofilm and it is suggested that the expression of PIA is a biomarker for biofilm formation [13]. The responsible regulator for PIA production are the *icaADBC* genes [14]. *SarA* directly activates the *icaADBC* locus. *SarA* also regulates the expression of superoxide dismutase. The locus *sodA* controls the superoxide dismutase in *Staphylococcus aureus*. When reactive oxygen species (ROS) are present, proteins, such as superoxide dismutase, are able to detoxify ROS and minimize cellular damage and, therefore, cellular stress [15]. 

With studies describing the alteration of implant surfaces after application of pressurized saturated steam, it may be necessary to investigate the influence of implant surface sterilization on bacterial behavior. Unexpected factors due to different sterilization methods can increase the pathogenesis of biofilm-forming bacteria. In this study, we examined the influence of different sterilization methods of titanium grade 4 and titanium grade 5 on the biofilm formation ability of *S. aureus*. We checked the gene expression of genes responsible for the biofilm formation on bacteria. The possible changes on material surfaces caused by sterilization can influence the ability of bacteria to form biofilms. Considering this information in the establishment of sterilization procedures for prosthesis could potentially help avoid periprosthetic joint infections.

## 2. Material and Methods

### 2.1. Titanium Material and Sterilization

We examined acid-etched titanium grade 5 (Ti6Al4V) and acid-etched CP grade 4 titanium (pure titanium) cylinders in this study. The samples had a cylindrical geometric form of 6 mm in height and 6 mm in diameter. The samples were polished and etched afterwards for 3 s with Kroll’s solution, containing 2.7% hydrofluoric acid, 5.5% nitric acid, and 91.7% H_2_O on the upper circular area of the cylinders. The institute for materials TU Braunschweig provided all titanium samples. Prior to biofilm formation, we washed the titanium samples in sterile distilled water, and then performed one of two different sterilization methods. Half of the used samples of titanium grade 5 and CP grade 4 were autoclaved at 121 °C for 20 min. The other half we sterilized with UV light for 1 h at 254 nm with 40 μW/cm^2^ intensity (ESCO SC2-6E1, UV-30A Lamp).

### 2.2. Biofilm Formation & Bacteria

In this study, we used *Staphylococcus aureus* ATCC 29213 as a biofilm forming strain. Firstly, we picked three colonies of the strain from a Mueller–Hinton agar plate, and suspended them in 2 mL of TSB + 1% glucose media in a 15 mL centrifuge tube (VWR international, Radnor, PA, USA). Afterwards, we incubated the pre-culture for 24 h at 37 °C on a shaker at 200 rpm. To guarantee biofilm formation only on the upper circular surface of the used cylinders, we fitted the titanium samples in silicone O-rings that sealed the space between the titanium sample and the used 48-well plate holes. We diluted the pre-culture at 1:100 with TSB + 1% glucose media and added 500 μL of *S. aureus* culture into each well. We incubated the cultures at 37 °C in a moist chamber on a shaker at 200 rpm for 24 h.

### 2.3. CFU

After biofilm formation, we removed the remaining media. Following this, we removed the titanium samples from the 48-well plates and washed them in PBS to remove planktonic bacteria cells. We added the samples into sterile vials. Then, we added 500 μL of TrypLE (Thermo Fisher Scientific, Waltham, MA, USA) to extract bacteria cells from the titanium surface. Before and after 5 min of incubation at 37 °C, we vortexed each vial for 15 sec. To inactive TrypLE, we added 500 μL of DMEM media. Before dilution steps were initiated, we vortexed each vial. We diluted each vial from 10^−1^ to 10^−8^ with TSB + 1% glucose and plated 10^−6^ to 10^−8^ dilutions in triplicates on MHA plates. The freshly plated MHA plates were incubated for 48 h at 37 °C. After incubation, we counted the CFU.

### 2.4. Primers

To evaluate expression levels of genes responsible for biofilm formation on CP4 and Ti6Al4V, we used the following genes and primers:
**Gene****Forward Primer****Reverse Primer***ica A*5′-CGC ACT CAA TCA AGG CAT TA-3′5′-CCA GCA AGT GTC TGA CTT CG-3′*ica B*5′-CAC ATA CCC ACG ATT TGC AT-3′5′-TCG GAG TGA CTG CTT TTT CC-3′*ica C*5′-CTT GGG TAT TTG CAC GCA TT-3′5′-GCA ATA TCA TGC CGA CAC CT-3′*ica D*5′-ACC CAA CGC TAA AAT CAT CG-3′5′-GCG AAA ATG CCC ATA GTT TC-3′*SarA*5′-AAG GAC AAT CAC ATC ACG AAG-3′5′-GAA CGC TCT AAT TCA GCG G-3′*Sod*A5′-GTT TCA TCA CGA CAA ACA TCA C-35′-TGA CAT CCT CAT CGC TTC C-3*SigB*5′-AGA AGC AAT GGA AAT GGG AC-3′5′-CTT AAA CCG ATA CGC TCA CC-3′*16s*5′-GAA AGC CAC GGC TAA CTA CG-3′5′-CAT TTC ACC GCT ACA CAT GG-3′

### 2.5. RNA Extraction

Following 24 h of biofilm formation, the samples were washed in PBS, and 6 samples of each titanium alloy and sterilization method were added into a 15 mL centrifuge tube (VWR international, Radnor, PA, USA). Then, 1 mL of TRI-Reagent (TRI Reagent^®^, Sigma-Aldrich, Saint Louis, MO, USA) was added. The tubes were vortexed for 15 s and sonicated for 3 min in an ultrasound bath (ultrasonic peak power: 800 W; Bactosonic, Bandelin electronic GmbH & Co. KG, Berlin, Germany). The liquids in the tubes were added to screw cap micro tubes (Sarstedt AG and Co., Nümbrecht, Germany) filled with 25–50 mg of glass beads (acid-washed glass globules, Ø 0.1 mm; Carl-Roth GmbH + Co., KG, Karlsruhe, Germany). The tubes were placed in a FastPrep-24TM 5G (MP Biomedicals, Thermo Fisher Scientific, Waltham, MA, USA) and processed three times for 35 s at 10 m/s. The tubes were cooled on ice for 2 min in between the repetitions. Afterwards, 200 μL of chloroform was added to each tube, the tubes were vortexed for 15 s, and incubated at room temperature for 7 min. Then, the tubes were centrifuged at 4 °C and 12,000× *g* for 15 min. Following the centrifugation, the upper phase containing the RNA was transferred to a 1.5 mL micro-centrifuge tube where an equal volume of 70% cooled ethanol was added. Afterwards, manufacturer’s procedures were followed as described in the QIAGEN Supplementary Protocol. For purification of total RNA from bacteria, RNeasy^®^ Mini Kit was used. The procedure was adapted to meet our requirements. The processed RNA liquid for each sample type was pooled on one membrane. The elution was performed with 50 μL of RNase-free water. For RNA quantification, 1 μL of the eluate was analyzed with a spectrophotometer (DeNovix DS-11 FX +μVolume Spectrophoto-/Fluorometer, Biozym Scientific GmbH, Hessisch Oldendorf, Germany). Next, 10 μg of extracted RNA were mixed with RNase-free water to achieve a total volume of 25 μL. Then, 1 μL of DNase I (2 units) with 2.6 μL of 10× Buffer from the kit were added. The first incubation was done at 37 °C for 30 min. Afterwards, 5 mM of EDTA was added to inactivate the reaction, followed by the second incubation at 75 °C for 10 min. After the DNase treatment, 1 μL of RNA was measured with a spectrophotometer.

### 2.6. cDNA Synthesis from Bacterial RNA

After RNA extraction, 1 μg of RNA was used for cDNA synthesis. We used the suggested procedure by the manufacturer (iScriptTM RT Supermix, Bio-Rad Laboratories, Feldkirchen, Germany). For performing the reaction protocol, we used the PikoReal 96 System (PikoReal 96 Realtime PCR System, Thermo Fisher Scientific, Waltham, MA, USA).

### 2.7. Bacterial Biofilm Gene Expression Using Real-Time Quantitative Polymerase Chain Reaction (Real-Time qPCR)

After we finished cDNA synthesis, we followed the protocol of the iQTM SYBR Green Supermix kit (iQTM SYBR Green Supermix, Bio-Rad Laboratories, Feldkirchen, Germany) with a total volume of 20 μL per reaction. For each real-time qPCR, 250 ng of each forward and reverse primer were used. Then we added 50 ng of cDNA. The same temperature and maximum times were used following the manufacturer’s procedures for thermal cycling (iQTM SYBR Green Supermix, Bio-Rad Laboratories, Feldkirchen, Germany). Default instrument settings were used for melting curve analysis (PikoReal 96 Realtime PCR System, Thermo Fisher Scientific, Waltham, MA, USA).

### 2.8. Statistical Analysis

For statistical analysis, we used GraphPad Prism 9. We calculated the CFU average with standard deviations and displayed these in bar graphs. We calculated gene expression fold change with delta-delta ct values for each replicate, and calculated standard deviation after we calculated the average for each gene and condition. We portrayed the results bar graphs with implemented deviations.

### 2.9. Scanning Electron Microscopy

To characterize the surface geometry of the titanium samples after UV-light sterilization and autoclavation, pictures of the surfaces were taken with a scanning electron microscope. The titanium cylinders were fixed onto aluminum pins using Leit-C gluing strips (Göcke, Plano GmbH, Wetzlar, Germany). The samples were then analyzed by scanning electron microscopy (SEM, JSM-6010LV, JEOL GmbH, Freising, Germany). 

## 3. Results

### 3.1. CFU

**Figure 1:**
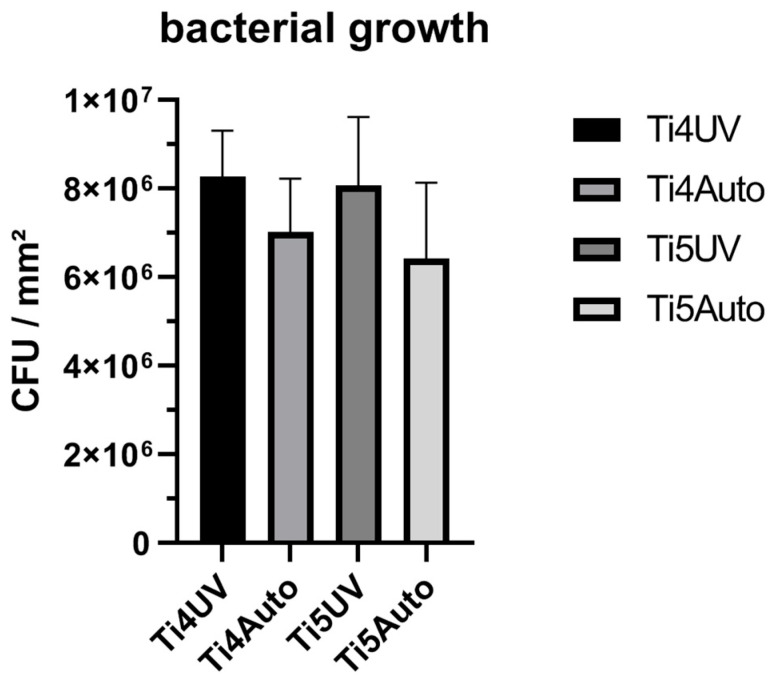
Colony forming units on UV light sterilized and autoclaved titanium grade 4 and titanium grade 5 samples.

Colony forming units of *S. aureus* on titanium grade 4 samples showed a higher tendency in colony counts when sterilized with UV light (Figure 1). Similarly, colony forming unit counts on titanium grade 5 samples were higher on UV light sterilized samples than on autoclaved samples. Overall, we detected no significant differences between growth on different materials nor different sterilization methods.

### 3.2. Gene Expression—Influence of Sterilization Methods on Biofilm Formation and Stress Induction

**Figure 2:**
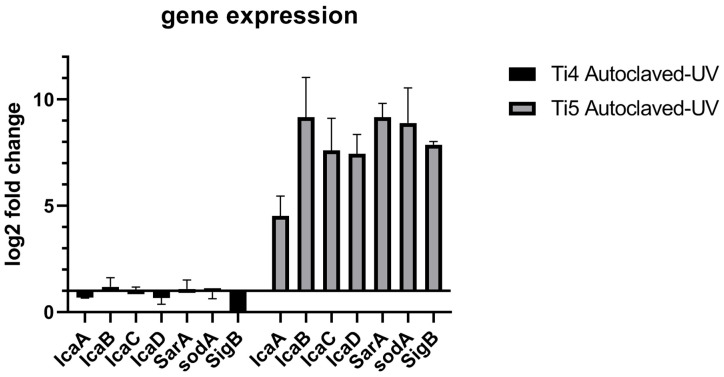
Comparison of log2 fold change of *IcaADBC*, *SarA*, *sodA,* and *SigB* between autoclaved to UV-light sterilized titanium samples.

Gene expression of *icaADBC*, *SarA* and *SodA* between autoclaved samples and UV light sterilized samples showed no difference on titanium grade 4 samples (Figure 2), whereas *SigB* showed higher gene expression on titanium grade 4 samples when sterilized with UV light compared to when steam-treated in an autoclave. On autoclaved titanium grade 5 samples, all examined genes showed 4–9 times higher fold changes in gene expression than on UV light sterilized samples.

### 3.3. Surface Characterization after Sterilization

**Figure 3:**
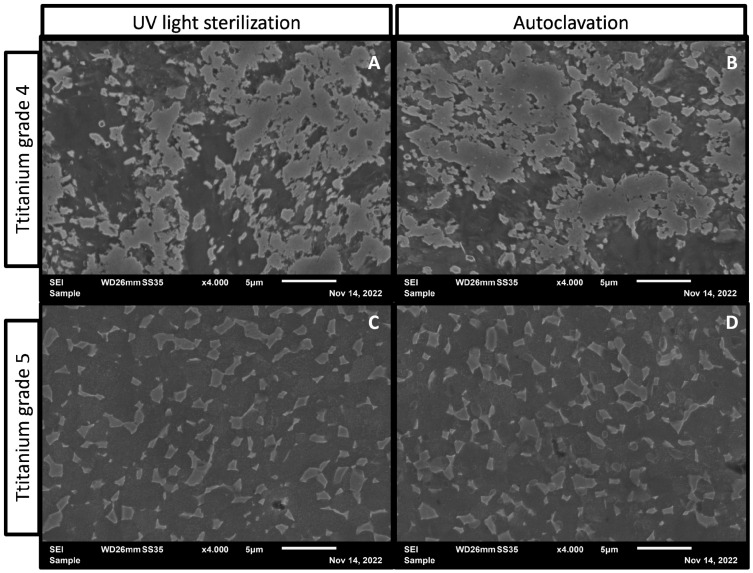
Sterilized titanium surface images performed by scanning electron microscopy. (**A**) Surface of titanium grade 4 sterilized with UV light; (**B**) surface of autoclaved titanium grade 4; (**C**,**D**) surface of titanium grade 5 samples after UV light sterilization and autoclavation.

Autoclaved titanium grade 4 samples (Figure 3B) showed small white dots on the titanium surface that are not present on the surfaces sterilized using UV light (Figure 3A), whereas on titanium grade 5, no white dots where visible after autoclavation. Nevertheless, after autoclavation of titanium grade 5, round forms are visible on the titanium surface (Figure 3D) that are not detectable on UV light sterilized titanium grade 5 (Figure 3C).

## 4. Discussion

The data obtained in this study showed significant higher gene expression in all examined genes of *S. aureus* ATCC 29213 on Ti6Al4V alloy after autoclavation and UV light sterilization. Higher expression of *icaADBC* genes indicates an increased formation of biofilm due to PIA production. *icaADBC* is regulated by *SarA*, which is directly regulated by *SigB* [14]. *SigB* is activated when cell stress occurs in *S. aureus* ATCC 29213. *SarA* and *SigB* gene expression are both significantly higher expressed (eight to nine times) on autoclaved Ti6Al4V in comparison to CP4. This may indicate that *S. aureus* was experiencing stress on the autoclaved Ti6Al4V titanium surfaces samples. The higher expression of superoxide dismutase *sodA* in *S. aureus* during our experiments indicates that ROS formation may play a crucial role in cellular stress on autoclaved Ti6Al4V.

To describe the effects on biofilm formation, which can be seen in this study, the mechanisms of UV light and steam on titanium surfaces must be understood. Titanium oxide (TiO_2_) has been reported to be a photo-catalyst during UV light exposure. The photo-catalytic effect of titanium oxide results in a biochemical formation of reactive oxide species (ROS) [16]. ROS are known to be a cellular stress factor in eukaryotic, prokaryotic, and inhibiting bacterial cells [17]. Nevertheless, Pan et al. reported that the photo-activation of titanium surfaces by UV light had a time dependent antibacterial effect, whereas Ranjan et al. reported the bacterial inhibition of photo-activated titanium by ROS [18,19]. The results in our study indicate a stronger tendency for autoclaved titanium surfaces to have an antibacterial effect. The non-significant lower numbers of CFU on both materials after autoclaving may be explained by material oxidations due to hydrothermal processes. Titanium is known for oxidation under hydrothermal conditions [20]. As shown in Figure 3, a visible surface modification occurs after autoclavation on both titanium alloys. Alloy compounds of CP4 and Ti6Al4V have shown antibacterial properties similar to titanium oxide, Al_2_O_3_, and vanadium oxide [21,22,23]. The significant increase of *sodA* expression on steam-treated Ti6Al4V can be explained by a possible increase of ROS formation on oxidized alloy compounds. It has been reported that titanium dioxide is able to increase ROS formation in bacterial cells and lead to cell death [24]. However, Hamida et al. had reported an inhibition of biofilm formation of a methicillin-resistant *S. aureus* strain under the induction of ROS [25]. 

This study suggests that the influence of ROS on biofilm formation is due to the possible oxidation of alloy components by the increased expression of superoxide dismutase proteins. One limitation of this study is the absence of results regarding the influence of ROS formation and ROS quantification in bacterial cells on autoclaved titanium alloys.

## 5. Conclusions

This study indicates that autoclaving Ti6Al4V can increase biofilm formation by *S. aureus* in vitro. The significantly increased gene expression of biofilm responsible genes may indicate a modification of titanium surfaces on alloy components. However, this study shows no significant differences between colony forming unit count between sterilization by steam or UV light on the titanium alloys studied. This study was able to show, that biofilm formation of *Staphylococcus aureus* is altered by steam sterilization on Ti6Al4V, which may lead to increased biofilm accumulation, increased horizontal gene transfer between cells, and therefore increased gene transfer of antibiotic resistance genes. It has been demonstrated, that autoclavation does lead to visible surface modifications. This study hints that steam sterilization of Ti6Al4V may lead to increased frequencies of antibiotic resistant *Staphylococcus aureus* genes, due to biofilm formation on steam-modified implant surfaces. Further studies must be performed to understand and confirm the oxidation and critical surface modification of Ti6Al4V alloy components.

## Data Availability

All data are presented in the publication.
